# Inferior vena cava evaluation in fluid therapy decision making in intensive care: practical implications

**DOI:** 10.5935/0103-507X.20190039

**Published:** 2019

**Authors:** Sofia Furtado, Luís Reis

**Affiliations:** 1 Departamento de Medicina Interna - Unidade 1.2, Hospital São José, Centro Hospitalar Universitário Lisboa Central - Lisboa, Portugal.; 2 Unidade de Urgência Médica, Hospital São José, Centro Hospitalar Universitário Lisboa Central - Lisboa, Portugal.

**Keywords:** Inferior vena cava, Echocardiography, Fluid therapy, Intensive care, Veia cava inferior, Ecocardiografia, Hidratação, Cuidados críticos

## Abstract

The fluid resuscitation of patients with acute circulatory failure aims to increase systolic volume and consequently improve cardiac output for better tissue oxygenation. However, this effect does not always occur because approximately half of patients do not respond to fluids. The evaluation of fluid responsiveness before their administration may help to identify patients who would benefit from fluid resuscitation and avoid the risk of fluid overload in the others. The dynamic parameters of fluid responsiveness evaluation are promising predictive factors. Of these, the echocardiographic measurement of the respiratory variation in the inferior vena cava diameter is easy to apply and has been used in the hemodynamic evaluation of intensive care unit patients. However, the applicability of this technique has many limitations, and the present studies are heterogeneous and inconsistent across specific groups of patients. We review the use of the inferior vena cava diameter respiratory variation, measured via transthoracic echocardiography, to decide whether to administer fluids to patients with acute circulatory failure in the intensive care unit. We explore the benefits and limitations of this technique, its current use, and the existing evidence.

## Abstract (pt)

A ressuscitação hídrica do paciente em falência circulatória aguda tem como um de seus objetivos aumentar o volume sistólico e, consequentemente, o débito cardíaco, para melhor oxigenação dos tecidos. Contudo, isso não se verifica em cerca de metade dos pacientes, que são considerados não respondedores a fluidos. A avaliação da resposta a fluidos antes de sua administração pode selecionar os pacientes que devem ter benefício e evitar o risco de sobrecarga nos restantes. Os parâmetros dinâmicos de avaliação da resposta a fluidos têm se revelado promissores enquanto fatores preditores. Entre estes, a medição ecocardiográfica da variação respiratória do diâmetro da veia cava inferior é um método de fácil aplicação, que tem sido difundido na avaliação hemodinâmica em unidades de cuidados intensivos. No entanto, a aplicabilidade desta técnica tem muitas limitações, e os estudos, até à presente data, são heterogêneos e pouco consistentes em alguns grupos de pacientes. Realizamos uma revisão sobre a utilização da variação respiratória do diâmetro da veia cava inferior, medida por ecocardiografia transtorácica, na decisão de administrar fluidos ao paciente em falência circulatória aguda, em cuidados intensivos, incluindo potencialidades e limitações da técnica, de sua interpretação e a evidência existente.

## INTRODUCTION

Patients with acute circulatory failure who exhibit signs of organ hypoperfusion and tissue hypoxia are common in the intensive care unit (ICU). The initial fluid resuscitation of patients in shock is associated with reduced mortality, and this effect is well established among patients with septic shock.^(1)^ However, after the initial fluid resuscitation phase, fluid administration is not necessarily beneficial.^(2)^ It might even be deleterious and lead to increased left ventricular filling pressure with pulmonary and tissue edema, which is associated with increased mortality and invasive mechanical ventilation (IMV) time.^(3)^

**Figure 1:**
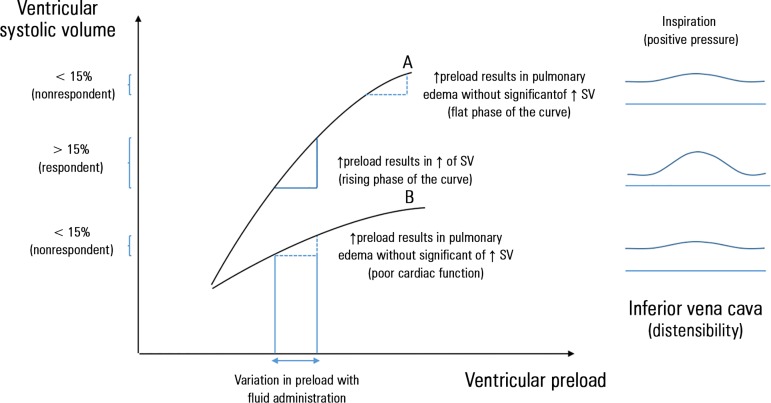
Frank-Starling curve and its relationship with inferior vena cava variation among patients under invasive mechanical ventilation. The relationship between preload and systolic volume: Frank-Starling curve. This figure shows the expected increase in systolic volume after the administration of fluids, which depends on cardiac function and the initial preload. For the same amount of fluids administered and for a similar initial preload, the variation in the resulting systolic volume depends on the cardiac function: (A) The Frank-Starling curve of a patient with normal cardiac function. In patients with normal cardiac function, the results of fluid administration only depend on the initial preload: If it is low (rising phase of the curve), then systolic volume significantly increases (≥ 10 - 15%, respondent patient), corresponding to a significant variation in the diameter of the inferior vena cava with the application of positive pressure to the thorax during inspiration in the ventilated patient; if it is elevated (flat phase of the curve), then no significant increase in systolic volume is observed (<10 - 15%, nonrespondent) leading to pulmonary overload, which corresponds to an inferior vena cava with little distension. (B) The Frank-Starling curve in a patient with decreased cardiac function. In this case, the administration of fluids, even with low initial preload, may result in pulmonary fluid overload without a significant increase in systolic volume. SV - systolic volume

Fluid therapy seeks to increase systolic volume (SV) and consequently improve cardiac output (CO) and oxygen transport to tissues. However, the fluid responsiveness of patients with shock is not linear because it depends on the contractile capacity of the myocardium (Figure 1). Since the contractile capacity is not directly measured and it is not possible to predict the configuration of the Frank-Starling curve of each patient, it is difficult to predict response to volume. Previous studies have shown that its administration does not result in increased CO in approximately 50% of patients.^(4-7)^

Therefore, the evaluation of fluid responsiveness aims to estimate the potential for a significant increase in CO in response to volume expansion, thereby avoiding inappropriate administration. A patient is considered a responder when an increase in CO of greater than 10 or 15% is observed;^(4,8,9)^ this result denotes that the patient is in the ascending phase of the Frank-Starling curve. In this patient, the administration of fluids would likely lead to increased SV, CO and consequent better oxygen delivery to tissues.

Several static and dynamic parameters have been evaluated as possible predictors of fluid responsiveness. In clinical practice, no standard reference or gold standard has been defined to evaluate fluid responsiveness; however, a growing consensus exists in favor of dynamic parameters^(2)^ because static parameters have shown no predictive value.^(10,11)^

The dynamic parameters are based on two ways of varying CO without administering fluids to predict the clinical response.^(4)^ One of these forms is through the lower limb elevation maneuver, which increases venous return and preload so that the CO variation is directly evaluated. The other form is based on the use of the lung-heart interaction. Variations in transpulmonary pressure with respiration induce CO variation, which is evaluated using one of the following: SV variation, variation in pulse pressure, variation in the diameter of the superior vena cava (SVC), or variation in the diameter of the inferior vena cava (IVC).

This article reviews the use of IVC respiratory variation to assess fluid responsiveness and its applicability among adults with acute circulatory failure in the ICU. We address the physiological principle, the technique, the clinical utility, the practical difficulties associated with its use and interpretation, and the underlying evidence.

## METHODS

A search was performed using the PubMed database with the terms "fluid responsiveness", "inferior vena cava", "echocardiography", "hemodynamic assessment", and "intensive care". The references of the included articles were also searched when considered relevant by the authors. The selection strategy was restricted to articles focusing on the use of IVC assessment among adult ICU patients published before January 1, 2018. No restriction filters were used with regard to language.

## DISCUSSION

### Echocardiographic evaluation of the inferior vena cava in the intensive care unit: Physiological principles, technique, and clinical indications

The assessment of IVC using transthoracic echocardiography is a conventional element of the echocardiographic study of critical patients. The physiological principle behind it is the lung-heart interaction. The variation in transpulmonary pressure during respiration is transmitted to the right heart cavities, which varies the venous return and the IVC diameter. This relationship depends on the ventilatory mode and IVC compliance of the patient.^(12-14)^

In nonventilated patients or those under IMV with respiratory effort, there is a negative transpulmonary pressure at the beginning of inspiration that induces a variable degree of IVC collapse as a function of its compliance. For example, in patients with high right heart cavity pressure or elevated preload (during the flat phase of the Frank-Starling curve), IVC shows reduced compliance and limited collapse due to the negative transpulmonary pressure transmitted; in fact, collapse may be absent. Among patients with low right heart cavity pressure in hypovolemia (i.e., the ascending phase of the Frank-Starling curve), IVC compliance is high, and collapse is significant during inspiration.

By contrast, positive pressure can be applied to the thorax during inspiration among patients under IMV without respiratory effort (in the controlled mode). This pressure is transmitted to the right heart cavities and the IVC, which stretches as a function of its compliance. Among patients without cardiac reserve due to poor cardiac function and/or those with high preload (i.e., during the flat phase of the Frank-Starling curve), the IVC shows reduced compliance and limited distention, and its diameter may not vary. Conversely, the IVC of patients with cardiac reserve who potentially benefit from the administration of fluids shows significant distension during inspiration.

**Figure 2:**
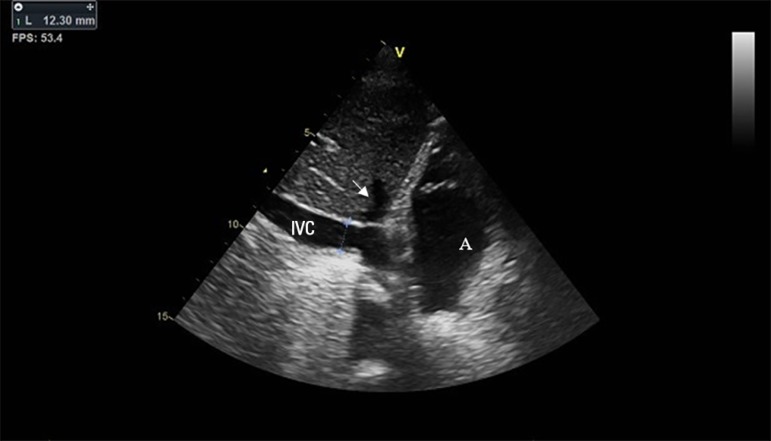
Inferior vena cava diameter measurement technique. The inferior vena cava should be measured in two-dimensional mode at the subcostal window using the long axis distal to the hepatic vein (arrow), approximately 1 - 3cm from the entrance of the inferior vena cava in the right atrium (A). Measurements near the right atrium entrance or near the diaphragm should be avoided. Its diameter can also be measured in M mode simultaneously with the two-dimensional mode to ensure the perfect alignment of the probe, perpendicular to the long axis of the inferior vena cava. In patients under invasive mechanical ventilation, the diameter of the inferior vena cava at the end of inspiration (maximal diameter) and at the end of expiration (minimum diameter) is measured to calculate the distensibility index. The probe must be kept in a fixed position during the respiratory cycle. Image obtained with a GE Vivid T8 echocardiograph. IVC - inferior vena cava.

At the technical level, the IVC diameter should be measured with the patient in the supine position at the subcostal window using the long axis. Measurements should be performed in the two-dimensional mode distal to the hepatic vein (i.e., approximately 1 - 3cm from the IVC entrance in the right atrium; Figure 2).^(15-17)^ The IVC diameter can also be measured in M mode, although a perfect probe alignment perpendicular to the long IVC axis is necessary. This measure implies the simultaneous use of the M and two-dimensional modes, with constant visualization of the IVC walls.

International recommendations^(17,18)^ suggest that the IVC be assessed to estimate the pressure in the right atrium of nonventilated patients because of its collapsibility during inspiration. An IVC diameter of < 21mm with a collapsibility of > 50% during inspiration suggests normal right atrium pressure (between 0 and 5 mmHg), whereas a diameter of > 21mm with collapsibility of < 50% suggests high pressure (between 10 and 20mmHg). A pressure between 5 and 10mmHg is considered intermediate; in this case, other parameters should be used to better characterize the pressure in the right atrium as normal or elevated, such as the size of the right atrium, hepatic flow, tricuspid regurgitation, and right ventricle function.^(17,18)^

**Figure 3:**
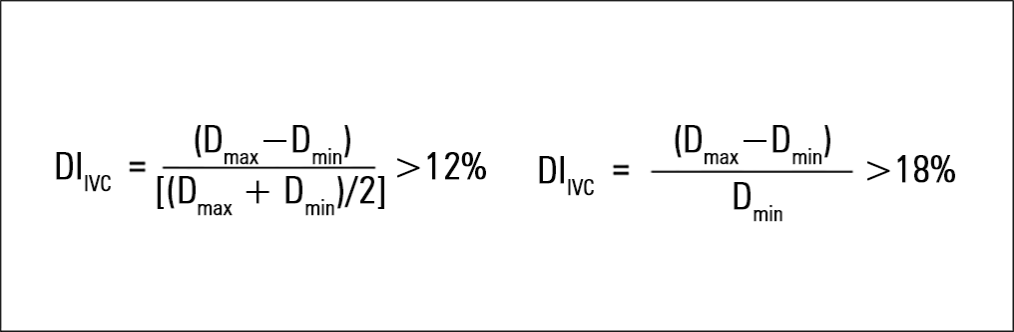
Formulas for calculating the distensibility index of the inferior vena cava. DI - distensibility index; Dmax - maximum diameter; Dmin - minimum diameter; IVC - inferior vena cava.

Inferior vena cava assessment can also be used to evaluate fluid responsiveness.^(18,19)^ At the end of expiration, an IVC diameter of < 10mm is frequent at low blood volume states, which suggests a higher probability of response, whereas a diameter of > 25mm is frequent during states of high blood volume and suggests a low probability of fluid responsiveness.^(20-23)^ However, these static values may not be relevant for the majority of patients, and their use is not indicated to predict fluid responsiveness because they do not show a reliable predictive value.^(9,24)^ On the other hand, the dynamic method of IVC evaluation, based on the variation in its diameter with respiration, enables the assessment of the potential benefit of fluid administration as a function of IVC compliance. However, the technique only showed a predictive value in a specific subgroup of patients: in IMV, in the controlled mode (without respiratory effort), with a tidal volume (TV) of ≥ 8mL/kg of ideal body weight.^(24,25)^ IVC diameter is measured at the end of inspiration (maximum diameter [D_max_]) and the end of expiration (minimum diameter [D_min_]) using transthoracic echocardiography, and the distensibility index is calculated using one of two possible formulas (Figure 3).

In the first formula, the distensibility index is considered significant (i.e., it predicts fluid responsiveness) if it is > 12%.^(24)^ The second formula predicts fluid responsiveness if it is > 18%.^(25)^

### Respiratory variation in the inferior vena cava as a predictor of fluid responsiveness: Limitations to its clinical applicability and interpretation

The use of IVC respiratory variation is limited for patients who are obese, laparotomized, or show a poor echocardiographic window^(13,26)^ because of limitations inherent to this technique that includes the use of echocardiography via the subcostal window. Its use also requires a correct application of the ultrasound probe for a reliable measurement. An incorrect echocardiographic measurement technique can lead to false-positive or negative results. For example, the incorrect alignment of the probe or lateral deviation of the IVC due to the pressure exerted by the probe at the abdominal wall can lead to a wrong measurement.^(26)^

Several limitations remain regarding the clinical applicability of this method; although not all of them have been studied, they should be considered.^(26)^ These limitations can be divided into factors that affect the variation in intrathoracic pressure, those that increase the right atrium pressure, and those that directly interfere with IVC compliance.

First, regarding the factors that affect intrathoracic pressure variation, attention must be paid to the positive end-expiratory pressure (PEEP) value and TV. A high PEEP value (e.g., during acute respiratory distress syndrome) elevates intrathoracic pressure, decreases the distensibility of the IVC, and leads to a false-negative result. Also, with a TV lower than 8 mL/kg, the pressure variations induced by IMV may not be sufficient to reliably vary the IVC diameter.^(13)^

The value of this technique is also questionable in ventilated patients with respiratory effort (IMV in the assisted or spontaneous mode). During the respiratory stimulus, a negative transpulmonary pressure exists that is the inverse to that caused by IMV. Therefore, it is not possible to control or predict the effective variation in the IVC diameter among these patients.^(26)^ In addition, in patients with respiratory stimuli (either ventilated or nonventilated), the respiratory pattern can vary, and the pressure variation in the chest will not be constant, leading to possible false positives or negatives.^(27)^

Second, in patients with cardiac pathologies that are obstacles to venous return, pressure increases in the right atrium and consequently distends the IVC. This state is not related to the blood volume state^(26)^ and occurs in patients with right ventricular dysfunction,^(28)^ severe tricuspid regurgitation, or cardiac tamponade.

Finally, since the IVC is located in the abdominal cavity, it is subjected to intra-abdominal and chest pressure. Patients with increased intra-abdominal pressure will have IVCs with decreased compliance, which can lead to false negatives in patients under IMV. Other mechanical factors must be considered, such as thrombosis or extrinsic IVC compression.^(26)^ The same problem arises in individuals under extracorporeal membrane oxygenation (ECMO) with one of the cannula in the IVC. In these cases, transthoracic echocardiography serves only to monitor cardiac function and the position of the cannula.^(29)^

### Evidence regarding the use of the inferior vena cava respiratory variation to predict fluid responsiveness in patients with acute circulatory failure in intensive care units

#### Studies in adults under invasive mechanical ventilation without respiratory effort (controlled mode)

The use of the IVC to predict fluid responsiveness has only been validated in small groups of patients (Table 1) and under well-defined conditions. Feissel et al.^(24)^ showed a distensibility index (D_max_ - D_min_)/[(D_max_ + D_min_)/2] of > 12% among 12 patients with septic shock in controlled-mode IMV and a TV of ≥ 8mL/kg, with a negative predictive value of 92% and a positive predictive value of 93%. Barbier et al.^(25)^ found similar results in a similar group of patients (39 with septic shock under IMV with a TV of ≥ 8mL/kg), in which an IVC distensibility index (D_max_ - D_min_)/D_min_ of > 18% had high sensitivity and specificity values (90%) and an area under the curve (AUC) of 0.91 (95% confidence interval [95%CI] 0.84 - 0.98). Although these studies show that the characteristics of IVC variation make it a good predictive test, its applicability is limited by the lack of generalizability, given the small sample size and the specificity of the clinical context.

**Table 1:** Major published studies regarding the use of IVC respiratory variation to predict fluid responsiveness in adult ICU patients with acute circulatory failure

	N	Type of ICU, shock, and ventilation	Exclusion criteria	Respondent definition	Discriminatory value	S/E PPV/NPV AUC*
**Ventilated patients**						
Feissel et al.^(24)^	23	M, septic shock, TV 8 - 10mL/kg	Hypoxemia with risk of death, right ventricular failure†	Δ ≥ 15% CO after fluids (8mL/kg hydroxyethylamide 6% for 20 minutes)	ΔdVCI > 12%	NPV 92%, PPV 93%
Barbier et al.^(25)^	39	MC, septic shock, TV 8.5mL/kg	Impossible to perform EchoTT	Δ ≥ 15% CI after fluids (7mL/kg of modified fluid gelatin 4% for 30 minutes)	ΔdVCI > 18%	S and E 90% (ASC 0,91; 0,84 - 0,98)
Charbonneau et al.^(30)^	44	MC, septic shock, TV 8 -10mL/kg	Hypoxemia with risk of death, right ventricular failure†, respiratory effort, arrhythmia, impossible to perform EchoTT	Δ ≥ 15% CI after fluids (7mL/kg of hydroxyethylamide 6% in 15 minutes)	ΔdVCI > 21%	S 38%, E 61% (AUC 0.43; 0.25 - 0.61)
Theerawit et al.^(31)^	29	M, septic shock, TV 8mL/kg	Arrhythmia, ascites, severe valvulopathy or intracardiac shunt, contraindication to sedatives/anesthetics	Δ ≥ 15% CO§ after fluids (1 L 0.9% NaCl for 1 hour or 0.5L hydroxyethylamide 130/0.46% or 5% human albumin for 30 minutes)	ΔdVCI > 10%	S 75%, E 77% (AUC 0.69; 0.48 - 0.9)
Vignon et al.^(32)^	540	MC, shock of any cause, TV < 8mL/kg in 66%	Pregnancy, amputation, or severe ischemia in lower limbs, contraindication for TEE or LLEM¶	Δ ≥ 10% LVOT-VTI 1 minute after LLEM	ΔdVCI ≥ 8%	S 55%, E 70% (AUC 0.64)
**Nonventilated patients**						
Airapetian et al.^(9)^	59	MC, shock of any cause	Signs of bleeding, arrhythmia, compression stockings, contraindication to LLEM¶, immediate need of volume	Δ ≥ 10% CO after 0.5L of saline solution for 15 minutes	ΔcVCI > 42%	S 31%, E 97% NPV 59%, PPV 90% (ASC 0,62; 0,49 - 0,74)
Muller et al.^(27)^	40	UN, septic, hemorrhagic, hypovolemic shock	Pulmonary edema, right ventricular failure or high RA pressure†	Δ ≥ 15% LVOT-VTI after 0.5L hydroxyethylamide 130/0.46% for 15 minutes	ΔcVCI > 40%	S 70%, E 80% (ASC 0,77; 0,60 - 0,88)

* With 95% confidence interval when reported in the literature;

† documented by transthoracic echocardiography;

§ cardiac output was obtained from FloTrac/Vigileo (third generation), which is not the gold standard for assessing CO;

¶ for example, elevated intracranial pressure, cardiac tamponade, and acute aortic dissection;

ICU - intensive care unit; S - sensitivity; E - specificity; PPV - positive predictive value; NPV - negative predictive value; AUC - area under the curve; M - medical; TV - tidal volume; CO - cardiac output; dVCI - distensibility index of the IVC; MC - medical-surgical; EchoTT - transthoracic echocardiography; TEE - transesophageal echocardiography; CI - cardiac index; NaCl - sodium chloride; LLEM - lower limb elevation maneuver; LVOT-VTI - time-velocity integral of the left ventricular outflow tract; cVCI - collapsibility index of the IVC; UN - unspecified; RA - right atrium.

Additional studies^(30,31)^ of patients in septic shock had less consistent results, showing discriminatory powers of AUC = 0.43 (95%CI 0.25 - 0.61) and AUC = 0.69 (95%CI 0.48 - 0.89), respectively. One potential explanation of this discrepancy compared with previous studies is related to the fact that Charbonneau et al.^(30)^ found a higher percentage of patients receiving laparotomy (23% versus 9% in Barbier et al.^(25)^^))^, which might have conditioned the accuracy of the test casting doubts about its use among patients undergoing abdominal surgery. In the case of Theerawit et al.,^(31)^ patients with severe sepsis were included, who might have increased intra-abdominal pressure in that context. Intra-abdominal pressure was not monitored and may have biased the results.

More recently, a study with a larger and more heterogeneous sample revealed less promising results. Vignon et al.^(32)^ conducted a prospective, multicenter study of 540 patients with circulatory failure of any cause and under IMV. They compared the respiratory variation in SVS, IVC, and maximal aortic velocity with the test of lower limb elevation (i.e., the standard reference). In this study, only 42% of the patients responded to fluids, and the variation in SVS was the best discriminatory test. However, this finding implies the use of transesophageal echocardiography, which limits its applicability. The index of IVC variation exhibited a 55% sensitivity (95%CI 50 - 59) and a 70% specificity (95%CI 66 - 75). However, the discriminatory value considered was 8%, and the assessment of the IVC was only possible for 78% of patients due to the difficulties in image acquisition because of recent surgery (approximately 25% of the patients), which might have reduced its diagnostic acuity. Furthermore, most patients had a protective ventilatory mode with TV < 8mL/kg, which was contrary to previous studies. Despite these limitations, this sample was larger than those of other studies and included multiple causes of shock, which reflects usual clinical practice conditions and their inherent limitations. The authors concluded that the discriminatory power of these parameters was not sufficient to overcome clinical judgement and recommended fluid bolus if the risk is low and signs of hypoperfusion are present, even if the echocardiographic parameters predict a weak response.

#### Studies in nonventilated adults

Studies have shown high specificity but low sensitivity with regard to nonventilated patients in the ICU. Muller et al.^(27)^ showed that in 40 nonventilated patients with hemorrhagic, hypovolemic or septic shock, an IVC collapsibility index of > 40% had a specificity of 80% and a sensitivity of 70%, with an AUC of 0.77 (95%CI 0.60 - 0.88); however, the test was not reliable concerning these patients because the lower limit of the 95%CI of the AUC was < 0.75. An IVC collapsibility index below 40% does not allow us to exclude fluid responsiveness, and the probability of response increases when the index is above 40%. Airapetian et al*.*^(9)^ found similar results among 59 nonintubated, nonventilated patients, in which a collapsibility index of > 42% had a specificity of 97% and a positive predictive value of 90% but low sensitivity and negative predictive values, with an AUC of 0.62 (95%CI 0.49 - 0.74).

The main characteristics of the aforementioned studies are shown in table 1. These studies are highly heterogeneous; thus, comparisons are difficult. A standard reference (namely, the parameter considered [cardiac index, CO, or SV index], the maneuver used, the type of fluid administered, or its mode of administration) does not exist with regard to the definition of a fluid responder, which is a limiting factor in the study of this technique.

#### Meta-analyses of the use of inferior vena cava respiratory variation to predict fluid responsiveness in intensive care units, regardless of ventilatory mode or clinical context

In a 2014 meta-analysis^(29)^ of eight studies including 235 patients, either nonventilated or under IMV, the combined sensitivity was 76% (95% CI = 61 - 86) and the specificity was 86% (95%CI 69 - 95). The combined AUC was 0.84 (95%CI 0.79 - 0.89). The discriminatory value of IVC variation ranged between 12 and 40% across these studies. Of the patients under IMV, better sensitivity (81%; 95%CI 67 - 91) was found for similar specificity (87%; 95%CI 63 - 97). In a 2017 systematic review and meta-analysis^(7)^ of 17 studies including 533 patients with circulatory failure, the combined sensitivity and specificity values of the IVC variation index to predict fluid responsiveness were 63% (95%CI 56 - 69) and 73% (95%CI 67 - 78), respectively, with a combined AUC of 0.79 (standard error = 0.05). The subgroup of ventilated patients (combined sensitivity = 67% [95% CI = 58 - 75]; specificity = 68% [95%CI 60 - 76]) presented with better results than nonventilated patients (combined sensitivity = 52% [95%CI 42 - 62]; specificity = 77% [95%CI 68 - 84]) as previously shown. The authors reported that the respiratory variation in the IVC diameter moderately predicted fluid responsiveness and that a negative test does not exclude fluid responsiveness; thus, its clinical usefulness is limited, particularly among nonventilated patients. Because these meta-analyses include original studies across varied clinical contexts (ICUs and emergency departments; type of circulatory shock and ventilation; mode of measurement of IVC; the considered discriminatory value or standard reference), their results should be valued accordingly. The value of the IVC test depends on the clinical context, which should be considered during assessment and interpretation.

### Use of inferior vena cava respiratory variation to evaluate fluid responsiveness in clinical practice in intensive care units: advantages, disadvantages, and current outlook

The use of IVC variation is favored among the dynamic methods of fluid responsiveness assessment in the ICU because it is noninvasive, inexpensive, easy, and reproducible; moreover, it does not demand a high level of training.^(33,34)^ In addition, complementary echocardiographic assessment, both quantitative and qualitative, contributes to a better overall clinical evaluation.^(8,18)^

However, the use of IVC regarding the decision to administer fluids should be considered only if certain technical and clinical conditions are met, i.e., patients under IMV, in the controlled mode (without respiratory effort), TV ≥ 8mL/kg, normal intra-abdominal pressure, and without acute cor pulmonale or severe right ventricular dysfunction. Otherwise, the studies are too heterogeneous and unlikely to be generalized.

The specificity of these conditions restricts the use of IVC respiratory variation.^(35-37)^ The studies evaluating the prevalence of the ventilatory conditions required for the application of this technique in the ICU (i.e., the prevalence of patients under controlled-mode IMV with TV ≥ 8mL/kg) show that these are present only in a small percentage of patients. In these studies, the possibility of a transitory increase in TV only to perform the maneuver was not considered. The various aspects limiting the use of IVC respiratory variation to predict fluid responsiveness may be one of the reasons for its infrequent use in the ICU.^(37)^ In the observational and multicenter study FENICE,^(37)^ which evaluated the way that physicians apply volume expansion among critically ill ICU patients, hemodynamic variables were used to predict fluid responsiveness in only 57.3% of the patients, of whom only 9.3% corresponded to echocardiographic parameters.

## CONCLUSION

Fluid therapy increases cardiac output in only approximately half of patients with acute circulatory failure. Ideally, patients with acute circulatory failure should be evaluated with regard to fluid responsiveness before its administration to avoid deleterious effects. In intensive care units, the use of inferior vena cava respiratory variation measured by transthoracic echocardiography may play a role in this evaluation; however, it is necessary to guarantee the conditions under which the technique is validated and to consider its limitations, depending on the clinical context, for correct interpretation. This technique has an unsatisfactory discriminatory power among nonventilated patients and those with respiratory effort because a negative test does not exclude fluid responsiveness.

The adequacy of resuscitation should be based on clinical judgment, considering the risk of fluid overload versus the potential benefit of fluid therapy, keeping in mind that not all responders need fluid administration. This practice must be individualized for each patient, integrating various clinical, echocardiographic, and biochemical parameters.
